# Additional treatment of vitamin D for improvement of insulin resistance in non-alcoholic fatty liver disease patients: a systematic review and meta-analysis

**DOI:** 10.1038/s41598-022-11950-x

**Published:** 2022-05-11

**Authors:** Dwijo Anargha Sindhughosa, I Dewa Nyoman Wibawa, I Ketut Mariadi, Gde Somayana

**Affiliations:** 1 Internal Medicine Residency Program, Udayana University/Sanglah General Hospital, Denpasar, Bali Indonesia; 2 Gastroenterohepatology Division, Department of Internal Medicine, Udayana University/Sanglah General Hospital, Denpasar, Bali Indonesia

**Keywords:** Liver, Liver diseases, Hepatology, Liver, Endocrine system and metabolic diseases

## Abstract

Insulin resistance provides an important role in the pathogenesis of non-alcoholic fatty liver disease (NAFLD). Several studies already evaluate vitamin D supplementation for NAFLD patients in relation to insulin resistance. The results obtained still carry conflicting results. This study aimed to evaluate the effect of additional treatment of vitamin D for the improvement of insulin resistance in NAFLD patients. Relevant literatures were obtained from PubMed, Google Scholar, COCHRANE, and Science Direct database. The obtained studies were analyzed using fixed effect model or random effect model. Seven eligible studies with a total of 735 participants were included. Vitamin D supplementation improves insulin resistance in NAFLD patients, marked by reduced Homeostatic Model Assessment for Insulin Resistance (HOMA-IR), with pooled mean difference − 1.06 (*p* = 0.0006; 95% CI − 1.66 to − 0.45). Vitamin D supplementation increase the level of vitamin D serum with pooled mean difference of 17.45 (*p* = 0.0002; 95% CI 8.33 to 26.56). Vitamin D supplementation decrease ALT levels, with pooled mean difference of − 4.44 (*p* = 0.02; 95% CI − 8.24 to − 0.65). No effect was observed for AST levels. Vitamin D supplementation provides beneficial effects on the improvement of insulin resistance in NAFLD patients. This supplementation may reduce HOMA-IR in such patients. It may serve as a potential adjunctive treatment for NAFLD patients.

## Introduction

Non-alcoholic fatty liver disease (NAFLD) is a spectrum of fat-associated liver conditions^1^. It is characterized by a high accumulation of triglycerides in hepatocytes, often accompanied with necro-inflammatory activity and fibrosis (steatohepatitis)^2^. It may develop to non-alcoholic steatohepatitis (NASH), fibrosis and cirrhosis. NAFLD has been known as a major cause of chronic liver disease, with increasing in prevalence, estimated as 25% to 30% among adults in developed countries^3,4^. Insulin resistance, inflammation and oxidative stress are considered as the main factor in the development of NAFLD^1^.

The pathogenesis mechanism of NAFLD is closely related to insulin resistance. Based on the most widespread model of the “two-hit hypothesis”, insulin resistance is involved in the process of the “first hit”. In this initial mechanism, it involves the accumulation of lipids located at the hepatocytes, in which insulin resistance is presumed as the main pathogenic factor for the development of hepatic steatosis. The “first hit” increase the vulnerability of the liver to factors that constitute the “second hit”. It may lead to hepatic injury, inflammation and fibrosis. The generation of proinflammatory cytokines, mitochondrial dysfunction, oxidative stress and lipid peroxidation also adipokines constituted as factors that may promote the development of hepatic injury^5^.

Vitamin D is a fat-soluble vitamin that regulates bone homeostasis. Its role has been widely explored, ranging to non-skeletal health diseases, e.g., metabolic syndrome, insulin resistance, obesity, type 2 diabetes, and cardiovascular-related disease. Recently, considerable scientific evidence explored the association of vitamin D and NAFLD. Vitamin D has been known to regulate insulin resistance, chronic inflammation, and fibrogenesis; hence vitamin D may benefit for preventing the progression of NAFLD^6^.

Several randomized controlled trials (RCTs) have evaluated the effect of vitamin D supplementation on insulin resistance. However, the results obtained still vary; either showed beneficial effects toward insulin resistance or showed no benefit^7–13^. Despite all conflicting results, meta-analysis to evaluate the overall effect of vitamin D supplementation is still required. Several meta-analyses had been done previously^14–16^. A meta-analysis by Guo et al. included six studies that assess the effect of vitamin D on insulin resistance provide substantial evidence that vitamin D may have a favorable effect on insulin sensitivity^14^. However, a different result was obtained by another meta-analysis. Pramono et al.^15^ found that additional vitamin D treatment showed no effect on insulin sensitivity. The population included in the study was subjects with or at risk for insulin resistance, not specifically targeted NAFLD population. Another study by Wei et al., which included four studies, also obtained similar finding. Vitamin D supplementation did not exert a reduction in HOMA IR^16^. Considering all the previous meta-analyses available regarding the use of vitamin D supplementation for insulin resistance, an updated meta-analysis with additional updated literature is needed. The objective of the current study aimed to evaluate the effect of vitamin D supplementation on insulin resistance.

## Results

### Study selection

**Figure 1:**
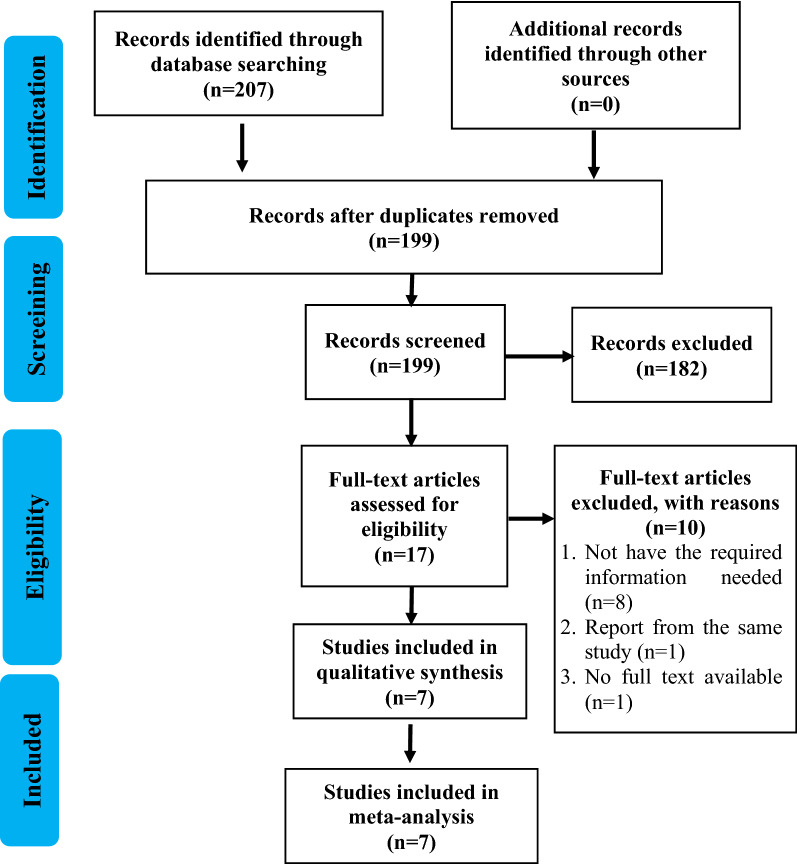
PRISMA flow diagram.

By utilizing the foremost search strategy, we found a total of 207 studies, and after the duplicates were removed, we obtained 199 articles. We excluded 182 articles by screening the titles and abstracts, left us a total of 17 relevant studies. Studies which not provide all the information needed for this meta-analysis or full-text not available were excluded. Screening and qualitative evaluation were performed, then we obtained seven articles used for the current systematic review and meta-analysis. PRISMA study flow diagram depicted in Fig. 1.

### Study characteristics

We included seven full-text articles of Randomized Control Trial (RCT). The publication year of these articles varied between 2012 and 2020, with a total of 423 samples for the intervention group and 312 samples for the placebo group. The experiment group received vitamin D supplementation in varying doses and duration, while the control group received placebo. The summary of findings and study characteristics can be seen in Table 1.

**Table 1:** Summary of findings and studies characteristics.

Author	Type of study	Population	Doses of vitamin D and comparator	Duration	Sample size, experiment versus control	Age, experiment versus control	Baseline HOMA-IR, experiment versus control	Baseline serum vitamin D, experiment versus control
Amiri et al.^7^	Randomized double blind placebo-controlled trial	NAFLD according to ultra-sonography	1000 IU supplement of vitamin D (25 μg/d as calcitriol; Jalinus Arya Co. Iran) Comparator: Placebo (25 μg/d as lactose; Jalinus Arya Co. Iran)	12 weeks	37 subjects versus 36 subjects	39.8 ± 11 versus 44 ± 10.8	4.3 ± 1.5 versus 3.55 ± 1.3	9.9 ± 3.9 versus 10 ± 3.8 ng/mL
Barchetta et al.^8^	Monocentric, randomized, double-blind, placebo controlled trial	T2D patients affected by NAFLD	Cholecalciferol 25.000 IU/2.5 mL) Comparator: Placebo; the recommended intake was eight drops a day, equivalent to cholecalciferol 2000 IU/day in the active-treated group	24 weeks	26 subjects versus 29 subjects	57.4 ± 10.7 versus 59.8 ± 9.1	3.57 ± 1.9 versus 3.87 ± 1.6	48.15 ± 23.7 versus 40.14 ± 23.9 nmol/L
Foroughi et al.^9^	Randomized controlled trial	NAFLD confirmed by ultrasound	Vitamin D3 50,000 IU every week Comparator: Placebo	10 weeks	30 subjects versus 30 subjects	The mean age of all participants was 48.5 years. No data o between group	3.1 ± 0.33 versus 3.12 ± 0.13	49 ± 1 versus 47 ± 2 nmol/L
Hussain et al.^10^	Double blind randomized placebo controlled trial	NAFLD patients by sonographic findings	Vitamin D3 50,000 IU orally weekly Comparator: Placebo	12 weeks	51 subjects versus 51 subjects	27 ± 1.7 versus 29 ± 19	4.56 ± 1.6 versus 4.32 ± 2.25	12.5 ± 4.2 versus 15.4 ± 2.82 ng/ml
Sakpal et al.^11^	Randomized controlled trial	NAFLD with normal or raised serum alanine aminotransferase (ALT)	Single injection of vitamin D (600,000 U) given intramuscularly Comparator: Lifestyle modifications *Note: all groups received standard medical treatment	6 months	51 subjects versus 30 subjects	37 ± 10 versus 40 ± 10	2.7 ± 2.1 versus 1.8 ± 1.6	12 ± 6.4 versus 12.3 ± 4.8 ng/dL
Sharifi et al.^12^	Randomized, double-blind, placebo-controlled trial with parallel design	NAFLD by ultrasonography scan and increased levels of alanine transaminase (ALT)	50,000 IU vitamin D3 (D-Vitin 50,000; Zahravi Pharm Co, Tabriz, Iran) every 14 days Comparator: placebo	4 months	27 subjects versus 26 subjects	40.33 ± 8.65 versus 43.92 ± 9.51	3.51 (2.61, 4.98) versus 2.52 (1.97, 3.32)^a^	11.50 (8.80, 28.40) versus 16.85 (11.70, 24.80) ng/ml^a^
Vesna et al.^13^	Randomized, double-blind placebo-controlled	Adult patients with NAFLD confirmed by ultrasound and transient elastography (TE)	Vitamin D3 oral solution (1000 IU/day; delivered as 5 drops, 200 IU each) Comparator: Placebo	12 months	201 subjects versus 110 subjects	64 (20–85) versus 66 (23–83)^b^	4.5 (0.45–146) versus 4.6 (0.68–121)^b^	59.3 (12.3–951) versus 47.3 (8.0–606) nmol/L^b^

HOMA-IR: Homeostatic model assessment for insulin resistance.

All data presented as mean ± SD, unless indicated otherwise.

^a^Data presented as median (Q1, Q3).

^b^Data presented as median (range).

### Risk of bias within studies

**Figure 2:**
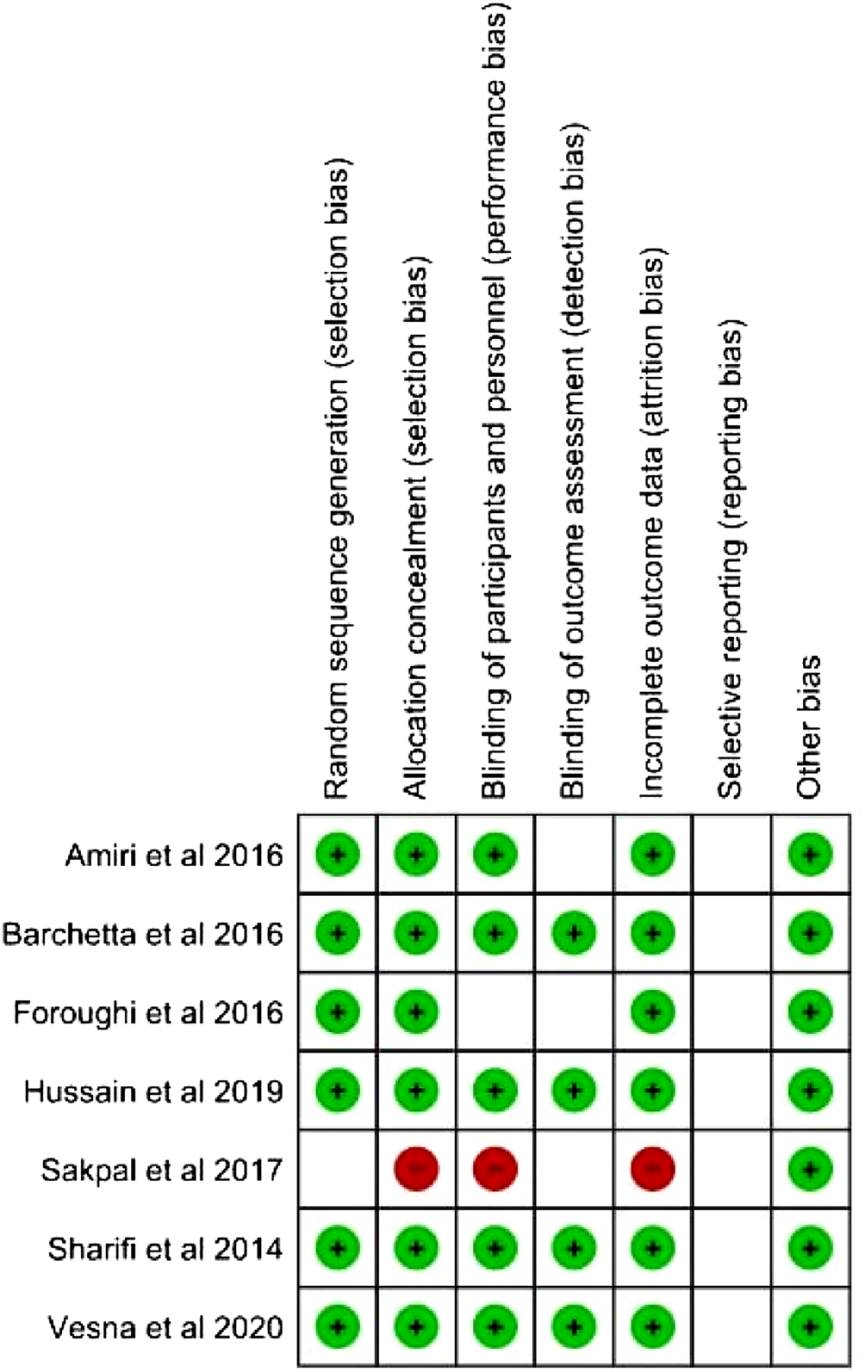
Risk of bias within studies.

The risk of bias was analyzed using Cochrane Collaboration’s risk-of-bias method. All seven articles included in the current study passed the evaluation of quality. The complete result of the risk of bias for all included articles described in Fig. 2.

### Effect of vitamin D supplementation on insulin resistance of NAFLD patients

**Figure 3:**
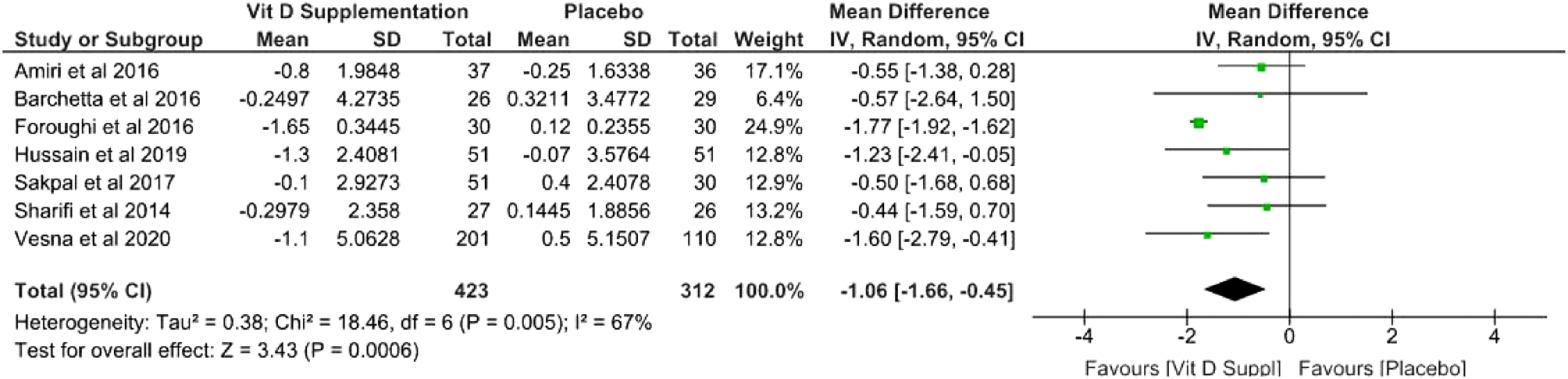
Forest plot of insulin resistance.

Vitamin D supplementation improve insulin resistance in NAFLD patients, marked by decrease of HOMA-IR. Based in random effect model (I^2^ = 67%; χ^2^ = 18.46; *p* = 0.005), pooled mean difference between vitamin D supplementation and without vitamin D supplementation was − 1.06 (*p* = 0.0006; 95% CI − 1.66 to − 0.45) (Fig. 3).

### Effect of vitamin D supplementation on serum vitamin D and liver enzymes of NAFLD patients

**Figure 4:**
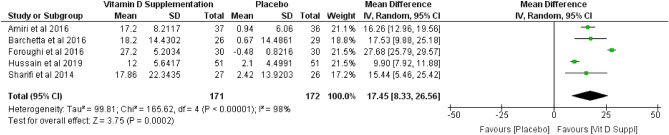
Forest plot of vitamin D serum.

**Figure 5:**
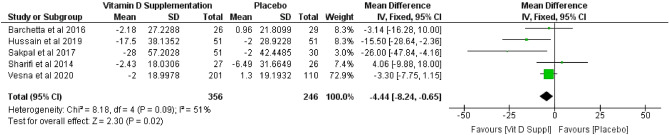
Forest plot of ALT levels.

**Figure 6:**
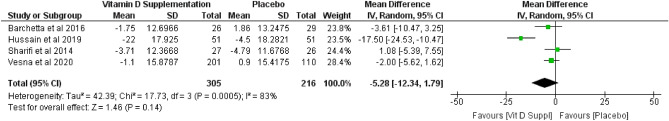
Forest plot of AST levels.

Pooled mean difference of vitamin D serum after vitamin D supplementation was 17.45 (*p* = 0.0002; 95% CI 8.33 to 26.56), based on the random effect model (Fig. 4). According to the analysis, vitamin D supplementation increase the level of vitamin D serum by 17.5 ng/mL. Meanwhile, the effect of vitamin D supplementation on liver enzymes of ALT and AST showed varying results. Vitamin D supplementation decrease ALT levels, with pooled mean difference of − 4.44 (*p* = 0.02; 95% CI − 8.24 to − 0.65) (Fig. 5). However, no effect was observed for AST levels, with a pooled mean difference of − 5.28 (*p* = 0.14; 95% CI − 12.34 to 1.79) (Fig. 6), based on random effect model.

### Meta-regression and sensitivity analysis

**Figure 7:**
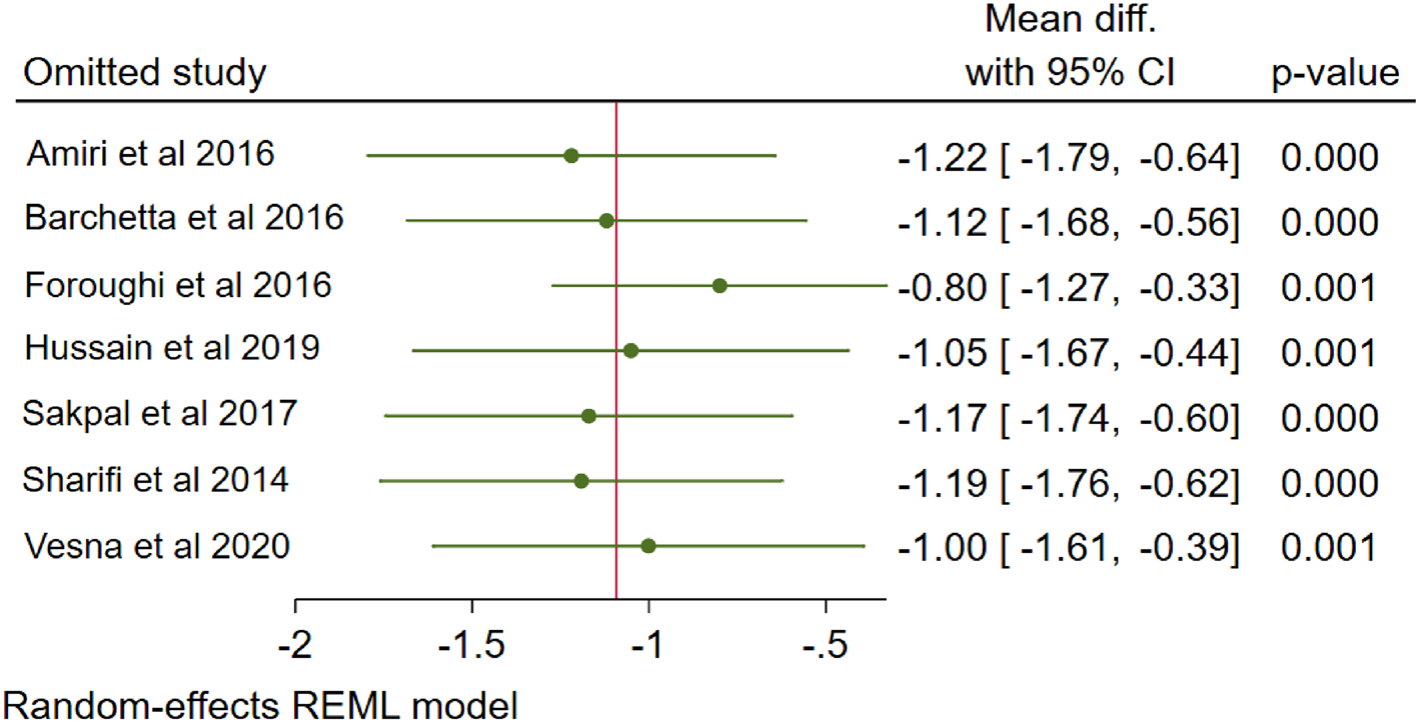
Sensitivity analysis by leave-one-out method, omitting each study.

A considerable heterogeneity (I^2^ = 67%) showed in regards to change in HOMA-IR following vitamin D supplementation. A meta-regression analysis of route of administration (oral or intramuscular), intake (daily or not), or duration of vitamin D supplementation (≤ 12 weeks and > 12 weeks) with mean difference of HOMA-IR showed that frequency of consumption may explain the heterogeneity (Table 2). Oral route of administration used in all study, except a study by Sakpal et al.^11^ Daily intake of vitamin D supplementation used in three studies^7,8,13^. Further sensitivity analysis by -leave-one-out analysis on the change in HOMA-IR following vitamin D supplementation showed that no study responsible for the heterogeneity of changes in HOMA-IR (Fig. 7).

**Table 2:** Result of meta-regression analysis with covariate of route of administration, intake and duration with HOMA-IR.

Covariate	Coefficient	SE	95% CI	*p* value	R^2^
Route of administration: oral	− 0.5992	0.7533	2.0756–0.8772	0.4263	0.98
Intake: daily	1.0958	0.4189	0.2747–1.9169	0.0089	
Duration: ≥ 12 weeks	0.6443	0.4117	0.1627–1.4513	0.1176	

*SE* Standard error of coefficient, *CI* Confidence interval, *R*^2^ R Square.

## Discussion

The pooled result of the current meta-analysis found that additional vitamin D treatment may improve insulin resistance, marked by decrease of HOMA-IR in patients with NAFLD. The route of vitamin D administration may vary, either intramuscular injection or oral. Its effect on improving insulin resistance was further analyzed for changes in serum ALT and AST levels. A decrement in ALT levels was observed due to additional vitamin D supplementation, but not for AST levels.

The occurrence of NAFLD is closely related to insulin resistance. Increased free fatty acid (FFA), adipose tissue inflammation and decrease of adiponectin are responsible for the development of insulin resistance in NAFLD^17^. Patients with NAFLD possess significant elevation of serum FFA, subsequently converted to triacylglycerol by the glycerol‐3‐phosphate pathway. The other product of this pathway is ceramides and diacylglycerols (DAGs). It has been known that DAG is involved in the activation of protein kinase C (PKC), which may inhibit insulin receptor threonine 1160, linked to reduced insulin resistance^18^﻿. Adipose tissue inflammation, along with the increase of pro-inflammatory cytokines such as interleukin 6 (IL‐6) and tumor necrosis factor alpha (TNF‐α), also lead to insulin resistance. As for adiponectin, it may promote fatty acid β‐oxidation (FAO), glucose utilization, and suppression of fatty acid synthesis. Its level decreases in NAFLD patients, hence promoting the development of insulin resistance^17^. In relation to vitamin D, vitamin D receptor (VDR) exists in hepatic cells and it is linked to decrease inflammation process in chronic liver diseases. The activity of VDR increases insulin sensitivity via modulation of FFA. Furthermore, vitamin D exerts anti-inflammatory and anti-fibrotic properties on the liver^19^.

Current evidence showed that vitamin D deficiency might be related to the pathogenesis of several diseases. This concept is true for the link between vitamin D deficiency and insulin resistance^20,21^. Vitamin D exerts its potential effect through its interaction with VDR and vitamin D-metabolizing enzymes. Those may be found in several cell types, including pancreatic β-cells and insulin-responsive cells such as adipocytes. Although the definite mechanism between vitamin D and insulin resistance is still uncertain, it has been suggested that adipose tissue may be related to its mechanism. The major vitamin D storage in the body is adipose tissue. It also served as a notable source of adipokine and cytokines, involved in the generation of systemic inflammation^22^. Current evidence suggested that vitamin D regulates the events involved in insulin secretion of pancreatic β-cells^23^.

Given such evidence, it is rational for additional vitamin D to improve insulin resistance in NAFLD patients. Recent reports pointed out the beneficial effect of vitamin D addition for improvement in insulin resistance. Several RCTs provide conflicting results, leading to the necessity of further evaluation through meta-analysis. A recent meta-analysis by Guo et al. to assess the effect of vitamin D on insulin resistance provides substantial evidence that vitamin D may have a favorable effect on insulin sensitivity. They found reduction in HOMA-IR by − 1.32; 95% CI − 2.30, − 0.34. The studies included for the evaluation of HOMA-IR were six studies^14^. However, conflicting evidence does exist. A systematic review and meta-analysis by Pramono et al., involving 18 RCTs, that evaluate the effect of vitamin D supplementation on insulin sensitivity in subjects with or at risk for insulin resistance showed that additional vitamin D treatment showed no effect of insulin sensitivity, with a standardized mean difference of − 0.01, 95% CI − 0.12, 0.10; *p* = 0.87, I^2^ = 0%^15^. However, it should be noted that the population evaluated in the meta-analysis was subjects with or at risk for insulin resistance (overweight, obesity, prediabetes, polycystic ovary syndrome [PCOS], and type 2 diabetes without complications), not NAFLD patients^15^. Another meta-analysis by Wei et al. also obtained similar finding. In the evaluation of vitamin D supplementation for HOMA-IR, which included four studies, vitamin D supplementation did not exert reduction in HOMA IR (WMD = 0.380, 95% CI − 0.162, 0.923; *p* = 0.169)^16^. Comparing all the data available, the current systematic review and meta-analysis provide more reports that vitamin D supplementation improves insulin resistance in NAFLD patients, similar to a meta-analysis by Guo et al. Although similar meta-analysis had been conducted, the current meta-analysis provides updated literature with more RCTs involved, hence providing stronger evidence for the effect of vitamin D supplementation on insulin resistance.

The effect of vitamin D on insulin resistance may be explained by its effect as a potential regulator of insulin secretion and Ca^2+^ levels. Calcitriol may directly trigger insulin secretion, since vitamin D responsive elements (VDRE) present in the insulin gene promoter, located at β-cells of pancreas^24^. Not only the transcription of insulin gene, VDRE is also known to stimulate various genes related to cytoskeletal formation, intracellular junctions and cellular growth of pancreatic c β-cells^25^. Vitamin D also showed an effect on insulin resistance through its regulation in Ca^2+^ flux. As calcium is essential for several insulin-mediated intracellular processes in muscle and adipose tissue, hence vitamin D may be related to its effect on insulin resistance. Optimal intracellular level of Ca^2+^ is mandatory for insulin action. Studies have found that vitamin D deficiency is responsible for increase concentration of Ca^2+^, leading to decreased activity of GLUT-4, impacting insulin resistance^26,27^.

The effect of improvement of insulin resistance due to vitamin D supplementation was further analyzed towards its effect on liver function, reflected by changes in ALT and AST levels. A decrement in ALT levels was observed due to additional supplementation of vitamin D, but not for AST levels. A meta-analysis by Guo et al. showed a borderline reduction in ALT levels and no effect toward AST levels, similar to this study^14^. Another meta-analysis study by Wei et al. in 2020 also found that serum alanine aminotransferase and aspartate aminotransferase levels did not differ between vitamin D supplementation and placebo groups^16^.

The current systematic review and meta-analysis also objected to limitations. The heterogeneity of the current meta-analysis may affect the results obtained in the present study. The future perspective should be directed to the number of studies and subjects involved in the evaluation of vitamin D supplementation toward insulin resistance, specified to the NAFLD population, and the homogeneity of the studies. Another aspect that needs to be considered is to involve other parameters in NAFLD to be investigated, for instance the effect of vitamin D supplementation in NAFLD patients toward inflammatory parameters, NAFLD activity score (NAS) and liver stiffness. To conclude, vitamin D supplementation improves insulin resistance in NAFLD patients, marked by the decrease of HOMA-IR. It may serve as a potential adjunctive treatment for NAFLD patients.

## Methods

### Eligibility criteria

The eligibility criteria were decided by implementing PICO concept. The framework depicted in Table 3.

**Table 3:** PICO framework of the study.

Patient	NAFLD patients
Intervention	Vitamin D supplementation
Comparator	Placebo
Outcome	Insulin resistance by HOMA-IR

### Type of studies

The current systematic review and meta-analysis included all studies prior to March 28th 2021 with full-text available, evaluating the additional administration of vitamin D in NAFLD patients. Articles with the type of case report, qualitative and economic studies, review, cadaveric and anatomic were excluded from the current study. All articles that did not provide the required data needed to conduct the current meta-analysis were also not included. To prevent the duplication of the sample, articles written by the same author within the same institution were evaluated for the samples.

### Type of participants

This review included studies with adult NAFLD patients who receive vitamin D administration. Insulin resistance was assessed using Homeostatic Model Assessment for Insulin Resistance (HOMA-IR).

### Type of intervention

The reviewed intervention is the administration of vitamin D. We included studies that administered vitamin D for any doses, any method of delivery and any duration. However, we noted both the doses and the duration of vitamin D given for each study.

### Type of outcomes

The primary outcome investigated in the current systematic review and meta-analysis was insulin resistance. In this regard, we use HOMA-IR to determine the insulin resistance of the patients. The secondary outcome includes serum vitamin D levels (ng/mL), alanine aminotransferase (ALT) (IU/l) and aspartate aminotransferase (AST) (IU/l) levels.

### Information sources

The eligibility criteria (PICO) were extracted into keywords utilizing Boolean operators (e.g., OR, AND, NOT) and all fields or MeSH (Medical Subject Heading) terms. In this study, we used keywords (NAFLD OR non alcoholic fatty liver disease OR NASH OR non alcoholic steatohepatitis) AND (Vitamin D OR Vitamin D3 OR Cholecalciferol OR ergocalciferol) AND (Insulin sensitivity OR HOMA-IR) in PubMed database, Google Scholar, COCHRANE, and Science Direct as search engine to find eligible journals.

### Study selection

The study selection process was conducted by three authors (DAS, IKM, GS) to minimize the likelihood of expunging the potentially relevant studies. When disagreement took place, the decision of the first, second and third authors was considered. Study selection started with disposing of duplicate records. Title and abstract screening were performed to exclude the irrelevant studies. Subsequently, studies that passed the first evaluation were further evaluated to evaluate their compliance with the inclusion and exclusion criteria for this review. All studies included were thoroughly assessed for its quality before eventually being included.

### Data collection process

All authors used an electronic data collection form to collect the required data from each of the articles. The data was then combined and managed with software Review Manager 5.4.

### Data items

The data items were the author’s name, year of publication, type of study, population, doses of vitamin D, duration of vitamin D administration, sample size, age, baseline HOMA-IR and baseline vitamin D levels. The mean difference of HOMA-IR after and before administration of vitamin D in both treatment and control groups for the respective duration were performed the meta-analysis.

### Assessment of quality of study

To ensure the quality of all articles which complied with the eligibility criteria for this review, a standardized critical appraisal tool was utilized. This process, which aimed to minimize the likelihood for bias in study selection, was performed independently by two authors (DAS and IKM).

The critical appraisal tool employed for this review was Cochrane Collaboration’s risk-of-bias method.

### Synthesis of result

The mean difference of HOMA-IR in NAFLD patients with administration and without administration of vitamin D was pooled and analyzed. If data presented as median with Q1 and Q3 or range, the mean was calculated using calculator, according to Luo et al. and Wan et al.^28,29^ The effect size reported as mean difference with 95% confidence interval (CI). Either fixed or random effect model was used for the analysis. Heterogeneity was assessed using the *I*^2^ statistic, indicating what proportion of the variation in observed effects across studies is due to the variation in true effects, with values > 60% indicating substantial heterogeneity. If the heterogeneity was > 60%, additional analyses were conducted with meta-regression analysis and sensitivity analysis. Sensitivity analysis conducted with leave-one-out method (removing one study each time and repeating the analysis). A *p* value of < 0.05 was considered significant. Meta-analysis was conducted using software Review Manager 5.4, sensitivity analysis performed with a statistical software package (Stata 17.0 for Windows) and meta-regression performed with Comprehensive Meta-Analysis Software Version 3.
